# Supplementary material for: Population genomic response to geographic gradients by widespread and endemic fishes of the Arabian Peninsula
Source: Ecol Evol. 2020 Apr 12;10(10):4314–30. doi: 10.1002/ece3.6199 (PMC7246217; doi:10.1002/ece3.6199)
Supplement: Supplementary file 6 — Appendix S1 [file ECE3-10-4314-s006.zip › Appendix S1, STRUCTURE and PCA Plots, Dryad/STRUCTURE/C. paucifasciatus/job_T13.pdf]

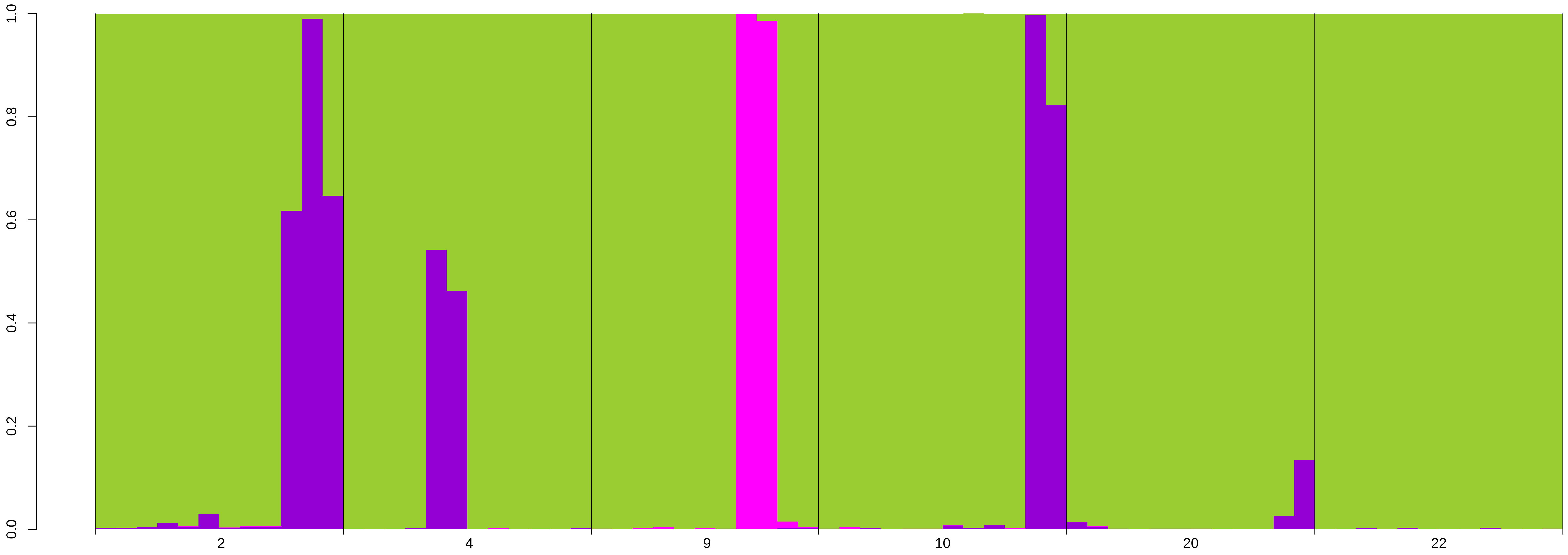

1.0
0.8
0.6
0.4
0.2
0.0
2
4
9
10
20
22
